# Evaluating the fundamental critical care support course in critical care education in Japan: a survey of Japanese fundamental critical care support course experience

**DOI:** 10.1186/2052-0492-1-5

**Published:** 2013-11-01

**Authors:** Kazuaki Atagi, Shinichi Nishi, Shigeki Fujitani, Takamitsu Kodama, Junya Ishikawa, Hideki Shimaoka

**Affiliations:** Division of Critical Care Medicine, Osaka City General Hospital, 2-13-22, Miyakojima-Hondori, Miyakojima, Osaka, 534-0021 Japan; Department of Intensive Care Medicine, Hyogo College of Medicine, 1-1 Mukogawa-cho, Nishinomiya Hyogo, Japan; Tokyo Bay Urayasu/Ichikawa Medical Center, 3-4-32 Todaijima, Urayasu, Chiba, 279-0001 Japan; Department of Surgery, Division of Emergency Medicine, The University of Texas Southwestern Medical Center, 5323 Harry Hines Boulevard, Dallas, TX 75390-9002 USA; Department of Emergency Medicine, Yokohama City University School of Medicine, 3-9 Fukuura, Kanazawa-ku, Yokohama, 236-0004 Japan

**Keywords:** Fundamental critical care support, Education, Simulator, Evaluation, Questionnaire

## Abstract

**Background:**

The Fundamental Critical Care Support (FCCS) course has been introduced after minimal adaptation according to Japanese clinical settings. The original course in the USA is often used to prepare residents for rotations in the intensive care unit (ICU). Therefore, the FCCS program can be appropriate for the basic training of critical care in Japan to standardize critical care management. The purpose of this study is to evaluate whether Japanese FCCS course is useful and has a possibility to deserve a basis of critical care management in Japan.

**Methods:**

The course program was provided with the form of lecture and skills stations. Pre- and post-training knowledge was assessed. After completion of the 2-day course, a questionnaire survey was administered to all course participants. Participants were asked to fill out the questions regarding socio-demographic characteristics. Participants were also asked to identify which lectures or skill stations they thought to be useful for clinical practice. Then, they were asked to rate their performance of each field: ‘Assessment,’ ‘Diagnosis,’ ‘Recognition,’ ‘Response,’ and ‘Transfer’.

**Results:**

The number of participants increased year after year and reached 1,804 during the past 4 years. Nearly 70% of the participants were physicians. Most of the others were nurses. In the established year, the percentage of physicians who had clinical experience more than 5 years exceeded 50%, however, this percentage gradually decreased. On the contrary, the percentages of residents and nurses increased. Regarding useful sessions, nearly half of the participants thought that mechanical ventilation was the most useful. With regard to the results of pre- and post-tests, the participants had already shown a high average mark (78.8 ± 14.1) at the pre-test. Furthermore, the score at the post-test was significantly improved (82.0 ± 6.6, *p* < 0.01). The participants' confidence in any field regarding critical care management was almost 4 points (5-point scale).

**Conclusions:**

It is considered that Japanese FCCS course is useful and has a promising basis of critical care management in Japan. Therefore, it is reasonable to think that Japanese FCCS mission has been successfully achieved.

## Background

Critical care training for medical personnel is crucial for the survival of the seriously ill patients. In addition, it is also important for improving critical care team performance including paramedical staffs to standardize and share critical care training program in accordance with the specific guidelines [1]. In Japan, however, the critical care training they receive is variable depending on the situation of the area or district. Besides, there is wide variation in the level of experience and qualifications of medical staffs in the intensive care units (ICU) throughout Japan because there are no specific requirements for employment beyond a valid general medical degree or something like that. Therefore, it is not unreasonable to suppose that the patient survival in the ICU is largely affected by staffs' expertise and commitment, although medical resources are almost equal at any ICU in Japan.

The Fundamental Critical Care Support (FCCS), a critical care training course developed by the Society of Critical Care Medicine (SCCM) in 1994, was launched in 1996 [2]. International SCCM members have worked as liaisons for its global distribution. Since February 2009, the FCCS course in Japan has been provided by Japanese Society of Education for Physicians and Trainees in Intensive Care (JSEPTIC) after minimal adaptation according to the Japanese clinical settings. Japanese FCCS committee provides a live, instructor-led 2-day course, which consists of the 13 lectures and their related skill stations like the original course. The purpose of FCCS is to train non-intensivists to manage critically ill patients for the first 24 h until transfer or appropriate critical care consultation can be arranged [3]. In addition, the course is often used to prepare residents for rotations in the ICU [3]. Therefore, the FCCS program can be appropriate for the basic training of critical care for intensivists including residents as well as non-intensivists in Japan to standardize critical care management.

Since the first Japanese FCCS course, a questionnaire survey has been conducted with participants of each course to assess the background of their participation and the impact of FCCS on critical care knowledge and performance of participants. The purpose of this study is to evaluate whether Japanese FCCS course is useful for improving knowledge and gaining confidence. Furthermore, a possibility that it deserves a basis of critical care management in Japan was also evaluated from 4 years of Japanese FCCS history.

## Methods

The first Japanese FCCS course was implemented in February 2009. Since then, 40 courses have been held and the number of participants reached more than 1,500 by December 2012. The course program was provided with the form of lecture and skills stations in accordance with the slide and curriculum licensed from the Society of Critical Care Medicine (SCCM). Temporal or spatial adaptation of the course agenda was arranged according to situations. The courses were open to anyone who was involved in critical care management. A representative course agenda in Japan is shown in the Table 1. Pre- and post-training knowledge was assessed using written multiple choice question (MCQ) examinations. The MCQ examination consists of 50 standardized questions developed by the SCCM and given in all FCCS courses. The typical MCQ involves a clinical scenario and often requires the interpretation of investigations. Rather than simply testing factual knowledge, the test is designed to test the ability of participants to make clinical decisions based on acute care scenarios. After completion of the 2-day course, a questionnaire survey was administered to all the course participants. Written consent was obtained from the participants before the survey. Names and any references made to individuals were de-identified to render the survey anonymous. Therefore, institutional review board approval was exempted because there were no ethical concerns. Participants were asked to fill out the questions regarding socio-demographic characteristics. Participants were also asked to identify which lectures or skill stations they expected to be useful for clinical practice before attending the course and felt to be surely useful for clinical practice after attending the course. Participants were also asked to rate whether their performance in each field met a desired standard and if this was the result of attending the course. The rating was made by a Likert scale of 1 to 5, indicating the extent to which participants agreed or disagreed to the statements. They are following items: Q1: prioritize assessment needs for the critically ill patient,Q2: identify appropriate diagnostic tests for the critically ill patient, Q3: recognize and initiate management of acute life-threatening conditions, Q4: identify and respond to significant changes in the unstable patient, and Q5: recognize the need for patient transfer and prepare the practitioners for optimally accomplishing transfer. To be able to do these items is the very goal for the FCCS course [3]. The questionnaire survey included other questions about evaluating the quality of each lecture or skill station and participant's requests for Japanese FCCS committee, etc. However, this study did not focus on these questions. Thus, the more details are omitted.

**Table 1:** **A representative FCCS course agenda**

Date	Time	Activity
October 21 (Monday)	7:30–8:00	Registration
	8:00–8:15	Pre-test/opening address
	8:15–8:20	FCCS overview
	8:20–8:50	Distinguish/assessment of critical ill patients
	8:50–9:30	Diagnosis and management of shock
	9:30–9:40	Break
	9:40–10:20	Monitoring blood flow, oxygenation, acid–base status
	10:20–11:00	Life-threatening infections: diagnosis and selection of antibacterial medicine
	11:00–11:40	Mental care
	11:40–12:20	Management of life-threatening electrolyte and metabolic disturbances
	12:20–13:30	Luncheon/instructor curriculum
	13:30–16:50	Skill station—day 1-, nos. 1–4, 50 min each, no break
		Shock
		Airway
		NPPV
		Line
	17:00–18:00	Acute coronary syndromes and special consideration
	18:00–18:40	Ethics in critical care medicine
	18:40–18:45	Question and answer
October 22 (Tuesday)	7:50–8:00	Pre-test review
	8:00–8:40	Diagnosis and management of acute respiratory failure
	8:40–9:20	Mechanical ventilation 1
	9:20–10:00	Mechanical ventilation 2
	12:20–13:00	Lunch time
	13:00–14:40	Skill station—day 2 B-, nos. 1 and 2, 50 min each, no break
		Shock 2
		MV
	14:40–15:00	Break
	15:00–16:00	Post-test
	16:00–17:00	Course evaluation

Summary statistics were performed using means, medians for asymmetrical distributed quantitative data, and proportions for categorical data. Statistically, comparison of pre- and post-training tests was tested using paired *t* test. To simplify statistical comparisons of the proportion changes of participants, participants were divided into two categories: the ‘Novice,’ 1–2-year residents and non-physicians; the ‘Expert,’ the others. Comparisons of proportion of the participants between years were tested using Chi-squared test. The improvement rate of the pre-test was calculated using the following formula: 100 (post-test − pre-test)/pre-test (%). The pre- and post-tests and the improvement rate of the pre-test between the Novice and the Expert groups were compared using Student's *t* test. Comparisons of the participants' confidence in the specific field were tested using Kruskal–Wallis test. Bonferroni's correction was used if needed for *post hoc* comparison. A *p* < 0.05 was considered as statistically significant.

## Results

**Figure 1:**
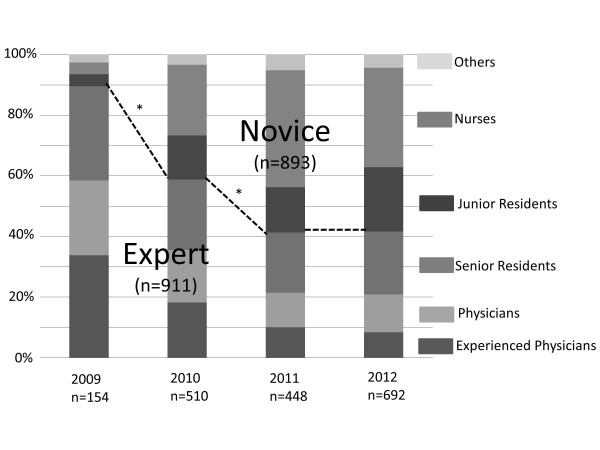
**Changes in the profession percentages of participants.** To simplify statistical comparisons of proportion changes of participants, participants were divided into two categories: the Novice, 1–2-year residents and non-physicians; the Expert, the others. Comparisons of proportion of the participants between years were tested using Chi-squared test. The percentages of novice participants statistically increased year by year. *p < 0.01 compared between years. Others: clinical engineers, physiotherapists, emergency medical technicians, dentists, and pharmacists. Nurses: any nurse. Junior residents: physicians who had clinical experience less than 2 years. Senior residents: physicians who had clinical experience more than 2 years and less than 5 years. Physicians: physicians who had clinical experience more than 5 years and less than 10 years. Experienced physicians: physicians who had clinical experience more than 10 years.

In 2009, when the Japanese FCCS course was launched, the number of participants of the courses was 154. By then, the number increased year after year and reached 1,804 during the past 4 years. Participants consisted of physicians, nurses, clinical engineers, physiotherapists, emergency medical technicians, dentists, and pharmacists. Nearly 70% of participants were physicians. Most of the others were nurses. Figure 1 shows changes in profession percentages of participants. In the established year (2009), the percentage of physician in the participants who had clinical experience more than 5 years exceeded 50%, however, this gradually decreased. On the contrary, the percentages of residents and nurses increased. The percentages of novice participants significantly increased year by year (*p* < 0.01); however, the percentages were not different between the last 2 years.

Regarding useful sessions, nearly half of the participants expected and surely felt that the session of mechanical ventilation consisted of two lectures and two skill stations was the most useful for clinical practice (Table 2). Even after dividing the participants into the two groups; the Novice and the Expert, both the groups showed the same tendency of responses about these questions (data are not shown).

**Table 2:** **Lectures or skill stations participants (** ***n*** **= 1,650) thought to be useful**

Title of the session	Before the course	After the course
	( ***n*** (%))	( ***n*** (%))
Neurologic support	7 (0.4)	8 (0.5)
Mechanical ventilation	752 (45.6)	799 (48.4)
Electrolyte and metabolic disturbances	21 (1.3)	26 (1.6)
Shock	182 (11)	186 (11.3)
Infections	50 (3)	26 (1.6)
Skill stations	89 (5.4)	68 (4.1)
Acute respiratory failure	27 (1.6)	20 (1.2)
Recognition/assessment of the seriously ill patient	31 (1.9)	16 (1.0)
Oxygen balance and acid–base status	53 (3.2)	55 (3.3)
Rapid response team	113 (6.8)	102 (6.2)
Critical care in pregnancy	4 (0.2)	5 (0.3)
Acute coronary syndromes	9 (0.5)	8 (0.5)
Airway management	16 (1.0)	17 (1.0)
Ethics in critical care medicine	5 (0.3)	9 (0.5)
Equipments	8 (0.5)	23 (1.4)
Others	28 (1.7)	46 (2.8)
All sessions	55 (3.3)	37 (2.2)
No answer	200 (12.1)	199 (12.1)

Participants in 2009 were excluded because these questionnaires were not included in the survey.

In regard with results of the MCQ examinations, the participants had already shown a high average mark (78.8 ± 14.1) at the pre-test. Furthermore, the score at the post-test was significantly improved (82.0 ± 6.6, *p* < 0.01). The average mark (75.5 ± 14.5) at the pre-test and (81.9 ± 7.1) at the post-test in the Novice group (*n* = 893) was significantly lower than the Expert group (*p* < 0.01, 83.3 ± 11.3 and 86.0 ± 5.7, respectively, *n* = 911). However, the improvement rate of the pre-test in the novice group was significantly higher than the expert group (12.2 ± 25.5% vs. 4.9 ± 16.1%, *p* < 0.01). Participants' confidence in any field regarding critical care management after course was almost 4 point (5-point scale) (Table 3). However, they felt less confident in the fields of ‘Diagnosis’ and ‘Transfer’ compared with ‘Assessment’ , ‘Recognition’ , and ‘Response’ (*p* < 0.01). Even between them, they also felt less confident in the matter of Transfer than Diagnosis (*p* < 0.01).

**Table 3:** **Participants' self-reported confidence to their performance of each field in critical care management after course (** ***n*** **= 1,666)**

	Assessment	Diagnosis*	Recognition	Response	Transfer* ^,^ **
Confidence scale	4.0 ± 0.1	3.8 ± 0.1	4.0 ± 0.1	4.0 ± 0.1	3.7 ± 0.2

Unfortunately, data from participants of the first several courses are missing. Properly speaking, data should have been presented by medians with range; however, means with standard deviation are used to instinctively realize the differences. Wilcoxon signed-rank test with Bonferroni's correction was used for statistical test

*p < 0.01 compared with Assessment, Recognition, and Response.

**p < 0.01 compared with Diagnosis.

## Discussion

In the first year, the Japanese FCCS course was launched with only 154 participants, who consisted of almost experienced physicians; however, the number of participants rapidly increased and professional backgrounds of participants were also dramatically changed. Recently, the percentages of junior residents and non-physicians constantly exceeded 50%. JSEPTIC was established in 2008 with the mission: the spread of education and research in the field of intensive care in Japan [4]. The Japanese FCCS course carries a major part of education in the JSEPTIC mission. Therefore, it is reasonable to suppose that early participants were mainly intensivists, who favored a philosophy of JSEPTIC. Their aim of attendance of the course was probably to learn what the standard education in intensive care but not the standard initial intensive care. However, the majority of recent participants are residents and non-physicians instead of physicians, who need basic critical care training. Thus, it is safe to say that the Japanese FCCS mission has been achieved. Recently, participating physicians with clinical experience more than 10 years are almost non-intensive physicians, who may encounter critical situations in their fields (data are not shown). The original policy of (FCCS) is to train non-intensivists for initial critical care management [3]. Thus, it is considered that the Japanese FCCS course starts to be also used as the original purpose of FCCS.

Regarding useful sessions, mechanical ventilation was the most attractive for participants. It is known that specific methods of mechanical ventilation management reduce mortality and lower health care costs [5]. However, it seems that there are few institutes which have a systematic educational program for mechanical ventilation. Even in the US internal medicine residency programs, only 46% of residents reported being satisfied with their mechanical ventilation training [6]. The FCCS course recognizes its importance and provides two lectures and two skill stations. Because the FCCS course, as a result, satisfied participant's request, mechanical ventilation may have gotten high expectations and satisfactions.

To achieve a score of 70% or higher on the post-test is one of requirements for successful course completion [3]. The average mark of the pre-test had already exceeded the requirement. Further improvement was also observed in the post-test, which was attributed to the course effects. The Japanese FCCS course has a relatively short history. Even at present, we cannot say that FCCS is already popular in Japan. Therefore, it can be considered that clinicians who had high motivation attended the course during this very dawning era. The average mark of the pre-test might decline in the future; however, we believe that the score of the post-test will be maintained at the same level due to this FCCS training. In addition, considering that the improvement rate of the pre-test in the Novice group was significantly higher than the Expert group, the Japanese FCCS course may be useful to improve participants' knowledge of critical care especially for the beginners. Participants' confidence in any field regarding critical care management after course was almost 4 point (5-point scale). Likert scaling is a bipolar scaling method, measuring either positive or negative response to a statement [7]. Consequently, they answered positive for all items. Therefore, it is safe to say that the goal of the Japanese FCCS course had been successfully accomplished. This is because being able to do these items (Assessment, Diagnosis, Recognition, Response, and Transfer) is the very goal for the FCCS course [3]. Of those items, course participants felt less confident in the fields of Diagnosis and Transfer compared with Assessment, Recognition, and Response. Even between them, they also felt less confident in Transfer than Diagnosis. Previously, it was reported that excessive ordering of tests and withholding information from the patients are the two examples of doctors' maladaptive responses to uncertainty with detrimental patient effects [8]. In addition, it has been suggested that the residents feel uncertainty in decision making at times of transition of care, specifically the determination of whether patients required escalation of care (e.g., transfer to the ICU) or were prepared for discharge [9]. Based on the above, it is natural to think that the obtained results were due to natural responses of trainees when they encounter critical incidents.

There are a couple of limitations to this study. First, the survey included responses from the participants in the very dawning era of the Japanese FCCS, who had high motivation as mentioned before. It is uncertain that the same results will be obtained with the upcoming generation in the future survey. Second, all questionnaires were self-reported. Therefore, a bias toward positive response by participants might have arisen because the early participants favored a philosophy of JSEPTIC. In addition, the results of participants' confidence in any field regarding critical care management might have represented only self-evaluation to their performance in clinical situations but not the impact of the course on their performance because the same self-evaluation was not performed before the course. Lastly, the Japanese FCCS course basically follows the original FCCS course in the US. However, the cultural and educational background in Japan is quite different from the US. Therefore, more adaptation or modification of the course might be required. In the future, the survey asking whether the current style of the FCCS course is suitable for the clinical situations in Japan may be conducted.

## Conclusions

Through this survey, it is considered that Japanese FCCS course is useful for improving knowledge and gaining confidence and that it is a promising basis of critical care management in Japan. Therefore, it is reasonable to think that Japanese FCCS mission has been successfully achieved. Participants until now have improved knowledge of critical care management and had positive confidence in implementing Assessment, Diagnosis, Recognition, Response, and Transfer, which is the goal of the FCCS after the course. In the next era of the Japanese FCCS, it is a challenge to provide the same success or more to the upcoming generation.
